# Reviewing the Peripheral and Central Mechanisms of Visceral Hypersensitivity in Intestinal Disorders

**DOI:** 10.7150/ijms.126361

**Published:** 2026-02-18

**Authors:** Wenhui Tang, Jiarui Wang, Wenlei Wang, Jiamiao Xue, Yuyan Wang, Fuhao Jiang, Dimkpa Christabel Kechiyerunda, Shengli Gao, Tao Yuan, Feifei Guo

**Affiliations:** 1 Physiology and Pathophysiology Department, School of Basic Medicine, Qingdao University, Qingdao, Shandong, China.; 2 Department of gastroenterology, Affiliated Qingdao Third People's Hospital, Qingdao University, Qingdao, Shandong, China.; 3 Special Medicine Department, School of Basic Medicine, Qingdao University, Qingdao 266071, China.; 4 Department of cardiology, Affiliated Qingdao Third People's Hospital, Qingdao University, Qingdao, Shandong, China.

**Keywords:** visceral hypersensitivity, dorsal root ganglion, nociceptor, gut microbiota

## Abstract

Visceral hypersensitivity (VH) is a condition where the internal organs have an enhanced sensitization to normal physiological stimuli or mild pathological stimuli, leading to chronic visceral pain or other discomforts, which is a typical characteristic of some intestinal disorders, such as irritable bowel syndrome (IBS) and inflammatory bowel disease (IBD). VH might be caused by gene, psychological disorders, social stress factors, gut microbiota, and some other factors, but the exact mechanisms are not yet clear. This review focuses on recent developments in the effect of intestinal cells on sensitization of nociceptors, high excitability of brain nuclei regulating visceral pain, and the novel roles of gut microbiota in VH. It is hoped to synthesize research advancements to demonstrate the possible peripheral and intracerebral processes of hypersensitization. Additionally, more animal experiments and clinical studies are still needed to improve our understanding about VH to reduce the suffering of patients with IBS and IBD.

## 1. Introduction

Visceral hypersensitivity (VH) denotes a heightened responsiveness and sensitivity of internal organs to different mechanical, chemical, and thermal stimuli, and often leads to typical symptoms including abdominal pain, bloating, altered bowel habits, and general discomfort in the abdominal region 1. Until now, this type of abdominal pain is difficult to manage, posing a challenge for patients and clinicians. The symptoms of VH can negatively impact daily activities, work performance, emotional well-being, and overall life satisfaction. VH is now recognized as a major pathophysiological mechanism contributing to abdominal pain in patients with functional or inflammatory bowel diseases, such as irritable bowel syndrome (IBS) and inflammatory bowel disease (IBD) 2. As known, IBD, including ulcerative colitis (UC) and Crohn's disease (CD), is characterized by marked mucosal inflammation, whereas IBS mainly demonstrates altered gastrointestinal environment, persistent low-grade inflammation, abnormal neuro-immune interactions, and dysbiosis 3, 4. Although IBS and IBD are two different diseases, IBD patients usually manifest IBS-like symptoms during remission, which is termed “irritable inflammatory bowel syndrome” for persistent hypersensitivity 5. Moreover, studies exhibited many overlapping influencing factors of IBS-like symptoms in IBS and IBD, such as sensitized sensory nervous system, enhanced immune responses, and impaired intestinal barrier 2.

It is known that VH might be caused by the sensitization of the afferent neural pathways located at different levels. Visceral nociceptors are mostly present at the nerve terminals of dorsal root ganglia (DRG) neurons, and can be directly activated by a wide variety of stimuli 6. For example, the transient receptor potential (TRP) can sense thermal, chemical, and mechanical stimuli. The voltage-gated sodium channels (Nav1.7, Nav1.8, and Nav1.9), Piezo1, Piezo2 can sense mechanical stimuli 7, 8. The purinergic receptors (P2X and P2Y), toll-like receptor 4 (TLR4), protease-activated receptor 2 (PAR2) and 5- hydroxytryptamine (5-HT) receptor can sense chemical stimuli 8, 9. The activated nociceptors potentiated action potential firing of DRG neurons, which project axons to the dorsal horn of the spinal cord. Then, the major nociceptive signals are sent to the contralateral ventral posterolateral nucleus of the thalamus via the spinothalamic tract. Neurons in thalamus relay the sensory information (pain) to the primary somatosensory cortex, posterior parietal cortex, and the limbic system for perceiving the pain signaling, processing emotional response, and making responding decisions 10. Based on the pathway for the transmission of visceral pain signals, VH arises primarily in the following processes: 1) sensitization of sensory neurons (predominantly involving changes in nociceptor function), 2) alterations in the excitability of spinal afferent pathways (with a focus on spinal dorsal horn neurons), and 3) sensitization of central pain-processing neurons. The exact causes of neural sensitization are not fully clear, but a complex interplay of intestinal cell function, gut microbiota, genetics, psychological factors, and environmental triggers might be involved.

Current narrative reviews on VH mainly focus on IBS. These reviews discuss advances in physiological mechanisms, covering potential drug targets like TRPV1, ASICs, voltage-gated sodium channels, ATP, PAR-2, cannabinoid, prostaglandin, tachykinin, and 5HT3 receptors 11. Research also shows that increased excitatory synaptic plasticity and changes in brain neural circuits contribute to VH 12, 13. Furthermore, the dynamic "microbiome shifts," characterized by the enrichment or depletion of specific bacterial taxa in IBS, and their significant impact on disease progression and pathology are explained 14. Recently, some articles have examined IBS-type symptoms in IBD, including low-grade residual inflammation, altered intestinal permeability, and VH, among others 15. The causes of VH in IBD are briefly outlined, such as highly sensitive or overexpressed TRPs, low-grade inflammation, alterations in the brain-gut axis, and post-inflammatory microbiota and metabolites 16-18. Thus, previous reviews primarily address pathophysiological mechanisms of VH, like abnormal nociceptors, dysbiosis, and enhanced sensory centers. However, few articles simultaneously discuss both IBS and IBD.

Based on the studies of VH in IBS and IBD, it is found that the peripheral nociceptors and central sensory neurons were strongly affected by molecules or transmitters from surrounding or functionally related cells, even the gut microbiota. For better understanding VH, this review presents the mechanism from three tightly interlinked aspects: (1) the active roles of intestinal cells (e.g., epithelial, enteroendocrine, immune cells) as translators of luminal stimuli into signals that prime peripheral nociceptor sensitization; (2) the specific circuits underlying the heightened excitability of defined brain nuclei, particularly those within the limbic and hypothalamic areas, which are responsible for maintaining the visceral pain state; and (3) the effects of gut microbiota dynamically influencing both peripheral intestinal afferent nerve and central sensory signals processing via the "microbiota-gut-brain" axis. This review integrates these components into a continuous narrative, tracing the pathogenic process from initial peripheral signal transduction in the gut to advanced central pain processing. We aim to provide a refined and coherent framework for understanding VH. Such an integrated perspective not only advances our mechanistic insight into the pathology of disorders like IBS and IBD but also illuminates novel potential targets for future therapeutic strategies.

## 2. Methods

### 2.1 Review design and guideline adherence

This narrative review designed to synthesize and critically evaluate current knowledge on the mechanisms of VH. To ensure methodological transparency and quality, the review was conducted and reported following the recommendations of the Scale for the Assessment of Narrative Review Articles (SANRA) 19.

### 2.2 Search strategy and information sources

A structured, broad literature search was performed to identify relevant studies. The search strategy was informed by key concepts related to our review's aim, loosely guided by a PICO framework:

Population/Problem: Patients with VH disorders (e.g., IBS, IBD with persistent pain) and relevant animal models.

Intervention/Exposure/Mechanism: Various biological mechanisms involve the interactions of intestinal epithelial cells, immune cells, the enteric and central nervous systems, and the gut microbiota.

Context: Pathophysiology and mechanistic research.

Electronic searches were conducted in the following databases: PubMed and Web of Science Core Collection. The search covered publications from inception until January, 2026. A combination of Medical Subject Headings (MeSH) terms and keywords was used, including but not limited to: “visceral hypersensitivity”, “visceral pain”, “irritable bowel syndrome”, “functional bowel disease”, “inflammatory bowel disease”, “intestinal barrier”, “enteroendocrine cell”, “mast cell”, “microbiota-gut-brain axis”, “TRPV1”, “central sensitization”, “stress”, “CRF”. Search strings were adapted for each database. The reference lists of key articles and recent reviews were also manually scanned to identify additional pertinent studies.

### 2.3 Eligibility criteria

Studies were considered for inclusion based on the following criteria:

Included: Original research articles (clinical, translational, and basic science), high-impact review articles, and meta-analyses published in peer-reviewed journals. We prioritized studies that elucidated mechanistic pathways in visceral pain perception, including human studies, rodent models, and seminal in vitro findings that inform biological plausibility. The focus was on literature published within the last two decades, with particular emphasis on high-impact findings and consensus views established in the field.

Excluded: Editorials, letters, conference abstracts without full data, studies exclusively focused on somatic pain pathways without visceral relevance, and articles not available in English.

### 2.4 Selection and synthesis process

The initial pool of records retrieved from database searches was deduplicated. The lead author screened titles and abstracts against the eligibility criteria to identify potentially relevant papers. Full-text versions of these articles were then obtained and assessed for final inclusion. Given the narrative and integrative nature of this review aimed at constructing a coherent model, the selection of representative literature was based on scientific rigor, novelty, and contribution to the overarching narrative of the gut-brain axis in VH. Conflicting results are recognized and explored in the text. The data from the included studies were thematically extracted and narratively combined to form the conceptual model in this review.

### 2.5 Evidence synthesis and grading

As part of our commitment to SANRA's emphasis on explaining the level of evidence, we implemented supplementary evidence grading framework. Key mechanistic statements in the text are tagged by primary evidence source (Human Clinical Study, Animal Model, *Ex Vivo*/*In Vitro*). Moreover, Table 1 provides a summary of the confidence and consistency of evidence for key pathways. This method improves the critical evaluation of the compiled literature.

## 3. Intestinal cells sensitizing sensory neurons

VH mainly arises from the abnormal activation of intestinal nociceptors. Some researches indicated that intestinal cells, such as intestinal epithelial cells (IECs), intestinal immune cells, and enteric glial cells (EGCs), directly sensitized nociceptors via different functional mediators.

### 3.1 IECs

The major cell types of the intestinal epithelium are enterocytes, enteroendocrine cells (EECs), Paneth cells, tuft cells, and goblet cells. In recent years, extensive researches have elucidated the role of the IECs on the DRG neurons in modulating visceral pain.

#### 3.1.1 Enterocytes

Enterocytes, as the main cell type in the intestine, play a key role in absorbing nutrients and secreting functional molecules like ATP, Glutamate (Glu), brain-derived neurotrophic factor (BDNF), IL-33, trypsin-3, and corticotropin-releasing factor (CRF).

ATP serves not only as the direct source of energy at the cellular level, but also as a signaling molecule to modulate the visceral sensation. After activated by heat, low osmolarity, mechanical stress, metabolites of arachidonic acid, and proinflammatory mediators via the TRPV4, enterocytes generate more ATP 112. Subsequently, excess ATP binds to P2X and/or P2Y purinoceptors to activate the nociceptors. For example, the P2X3 receptor subtype mediates the onset of pain sensitization by sensitizing TRPV1 expressed on DRG neurons or upregulation of β2 adrenergic signaling in primary sensory neurons, the P2X7 receptor subtype leads to increased release of IL-1β from immunocytes to indirectly induce VH, and the activation of P2Y receptors by ATP, UTP or ADP enhance the excitability of colonic pain sensation via the NaV1.9 channel co-expressed with P2Y on DRG neurons 20-22, 113.

Except ATP, the enterocyte activated by TRPV4 releases the excitatory neurotransmitter Glu. On the one hand, Glu directly excites the DRG neurons to participate in visceral pain 12, 25, on the other hand, Glu indirectly contributes to the visceral hypersensitive response through promoting the release of substance P (SP) and calcitonin gene-related peptide (CGRP) from DRG neurons and BDNF from enterocytes 12, 26-28, 114.

Furthermore, the enterocytes were found to secret more proteases and CRF in IBS patients and rodent IBS models. The proteases, predominantly trypsin-3, activate PAR2 of the enteric sensory neurons to induce VH 115. CRF indirectly contributes to VH by binding to CRF receptor 1 (CRFR1) on immune cells to promote inflammation via TLR4 and IL-1 pathways 116, 117. In both IBD patients and mouse models, the enterocytes secrete abundant IL-33, which subsequently acts on tumorigenicity 2 receptor (ST2) of mast cells. Activated mast cells disrupt the intestinal barrier and promote inflammatory responses, further contributing to the development of VH 41.

#### 3.1.2 EECs

Another important cell type of the intestine is EECs. Among various EECs, enterochromaffin cells (ECs), neuropod cells and L cells are believed to play more important role in VH.

##### 3.1.2.1 ECs

ECs are responsible for secreting approximately 95% of 5-HT in the body, which significantly influence intestinal movement and secretion 118. There are ongoing debates regarding the number of ECs in the patients with VH. Most research reported that patients with IBS showed a significant increase in ECs counts, and there was a significantly greater ECs number in patients with diarrhea-predominant IBS compared with patients with constipation-predominant IBS 32. A handful of studies reported that all IBS subtypes demonstrated a reduction in ECs 119, and there is still a small portion of research indicated that the quantity of ECs remains unchanged in IBS patients 36. Therefore, it is suggested that VH primarily attributed to alterations in ECs signaling rather than cell amount.

Studies suggested that individuals with IBS have notably elevated levels of 5-HT and 5-HT3R in the intestinal mucosal tissues compared to healthy people, and this abnormal overexpression was closely linked to the development of VH 34. Although the processes that elevate 5-HT release are still not completely defined, changes in intestinal pressure could play a role. Notably, mechanically gated ion channels of the Piezo family are expressed in intestinal epithelial cells 37, 38. Piezo1 and Piezo2 activate p38 signaling pathways, leading to increased expression of tryptophan hydroxylase 1 (TPH1), a key synthase for 5-HT in ECs, and thereby affecting 5-HT production 120. James et al. has proved that ECs establish synapses with intestinal mucosal afferent neurons to transmit visceral pain signals via the neurotransmitter 5-HT, which means more 5-HT releasing, higher visceral sensitivity 33. Besides, 5-HT might participate in VH by sensitizing TRPV1 and TRPV4 channels on DRG neurons 121, 122. All of the above suggests the pathway involving ECs, 5-HT, and mucosal afferent nerves (DRG) might play an important role in VH.

##### 3.1.2.2 Neuropod cells

Neuropod cells, as a specialized subtype of EECs with highly expressed Guanylyl Cyclase C (GUCY2C), modulates the DRG-neuron excitability via synapses 39. Clinical studies indicated that patients with IBS exhibit reduced levels of endogenous GUCY2C ligands, such as urinary guanosine 123. It is consistent with the animal experiment, which showed that blocking GUCY2C signaling leads to VH, and, conversely, activation of GUCY2C signaling alleviates visceral pain in colitis model mice 40. Although the detailed mechanism is still not clear, the basic process of GUCY2C signaling pathway has been explored. Ligand-activating GUCY2C converts guanosine triphosphate into cyclic guanosine monophosphate (cGMP), then cGMP acts as a second messenger to phosphate protein kinases, which leads to inhibit visceral pain transmission either by suppressing the release of excitatory neurotransmitters or by promoting the release of inhibitory factors 124.

##### 3.1.2.3 L cells

Another EECs L cells, were detected to secrete ATP, Glu, glucagon-like peptide 1, PYY and so on 24. ATP and Glu, like those released from enterocytes, can respectively act on P2X/P2Y receptors and glutamate receptors (N-methyl-D-aspartate receptors (NMDAR) and α-amino-5-hydroxy-3-methyl-4-isoxazolepropionic acid receptors (AMPAR)) located on neuronal endings, contributing to the onset of VH 125.

Table 2 summarizes intestinal epithelial cell-derived substances participate in VH by sensitizing nociceptors.

### 3.2 Immune cells

It is proved that VH of IBS and IBD patients is closely related to the activation of intestinal immune system, including enhanced infiltration of immunocytes, the low-grade inflammation, and so on. IBD is often characterized by infiltration of neutrophils, macrophages, innate lymphoid cells, mast cells, as well as Th1 (CD) and Th17 cells. Among these, neutrophils, macrophages, and mast cells play critical roles in the development of VH 127, 128. In contrast, mast cell and their close proximity to colonic nerves are more prominent in IBS, which is particularly evident in Diarrhea-predominant IBS-D and Post-infectious IBS (PI-IBS) 129. These immune cells contribute to VH primarily through two pathways: by releasing immune mediators that directly interact with nociceptive afferent neurons, and by altering intestinal permeability, thereby promoting VH 130, 131.

#### 3.2.1 MCs

Studies have found that the number of mast cells (MCs) is increased in the intestines of both IBD and IBS patients, which may be closely related to the occurrence of VH 132, 133. MCs, as major effector cells of innate immunity and regulators of adaptive immunity, secrete a wide array of inflammatory cytokines including IL-1β, IL-4, IL-5, IL-6, TNF-α and IFN-γ, as well as diverse inflammatory mediators such as histamine, tryptase and prostaglandin E2 (PGE2) 134.

The inflammatory cytokines not only act directly on the afferent nerve endings of DRG neurons to sensitize nociceptor ion channels, but also disrupt the intestinal barrier to facilitate the translocation of harmful substances across the barrier and exacerbate intestinal inflammation, which has been widely recognized as an important inducer of VH 135.

Many studies have showed that histamine released from MCs could induce VH via histamine receptor 1 (H1HR). D. Balemans et al. further explored that incubation of IBS colon biopsy supernatants or histamine-supplemented supernatants of healthy subjects with isolated mouse DRG neurons overnight enhanced neuronal Ca^2+^ responses by sensitizing TRPV1, TRPA1, and TRPV4 ion channels on DRG neurons, reducing the threshold for painful stimuli and inducing VH 58, 136. Additionally, the role of TRPV4 in histamine induced neuronal sensitization is more highlighted, because a MAPKK-dependent increase of TRPV4 expression on plasma membranes of colonic sensory neuron was closely related to H1HR relocation 58, 121.

Tryptases secreted from MCs can bind to the PAR2 on the afferent nerve endings of DRG neurons, potentially sensitizing TRPV1 of DRG neurons in a protein kinase C (PKC)-dependent manner to increase neuronal excitability 50. Likewise, the PAR2 of IECs is also recognized by tryptases, which triggers the release of intestinal inflammatory mediators 137. Furthermore, tryptases are found to cleave tight junction proteins, such as junctional adhesion molecule-A (JAM-A), claudin-1, claudin-3 and claudin-5, leading to intestinal barrier dysfunction 138, 139. All of these effects of tryptases contribute to VH.

PGE2 is another important inflammatory mediator released from MCs. In patients with IBS-D, lipopolysaccharide acting in concert with trypsin was found to stimulate mucosal mast cells to release PGE2 54. PGE2 binds to E-type prostanoid receptor 2 located on the afferent nerve endings of nociceptive DRG neurons, activating TRPV1 and various sodium channels 53. Moreover, a mouse model study has demonstrated that in patients with IBS-D, substances such as lipopolysaccharide and trypsin can stimulate mast cells to release PGE2, downregulate the serotonin reuptake transporter, and elevate mucosal 5-HT levels, thereby contributing to the development of VH 54.

Surprisingly, some neurotrophin and neuropeptides secreted by MCs are involved in the modulation of nociceptors. Nerve growth factor (NGF) also activates TrkA receptors on nerve endings to promote Nav1.7 currents and sensitize TRPV1 channels 140. Simultaneously, the neuropeptide CRF modulates the macrophages to release inflammatory cytokines such as IL-1 and IL-6, which would reduce the expression of the tight junction protein claudin-2 in the colonic mucosa leading to increasing intestinal permeability and inflammatory response 116, 141. Therefore, it is believed that the effects of neurotrophin and neuropeptides on neurons, macrophage and IECs would further contributes to VH.

**Figure 1:**
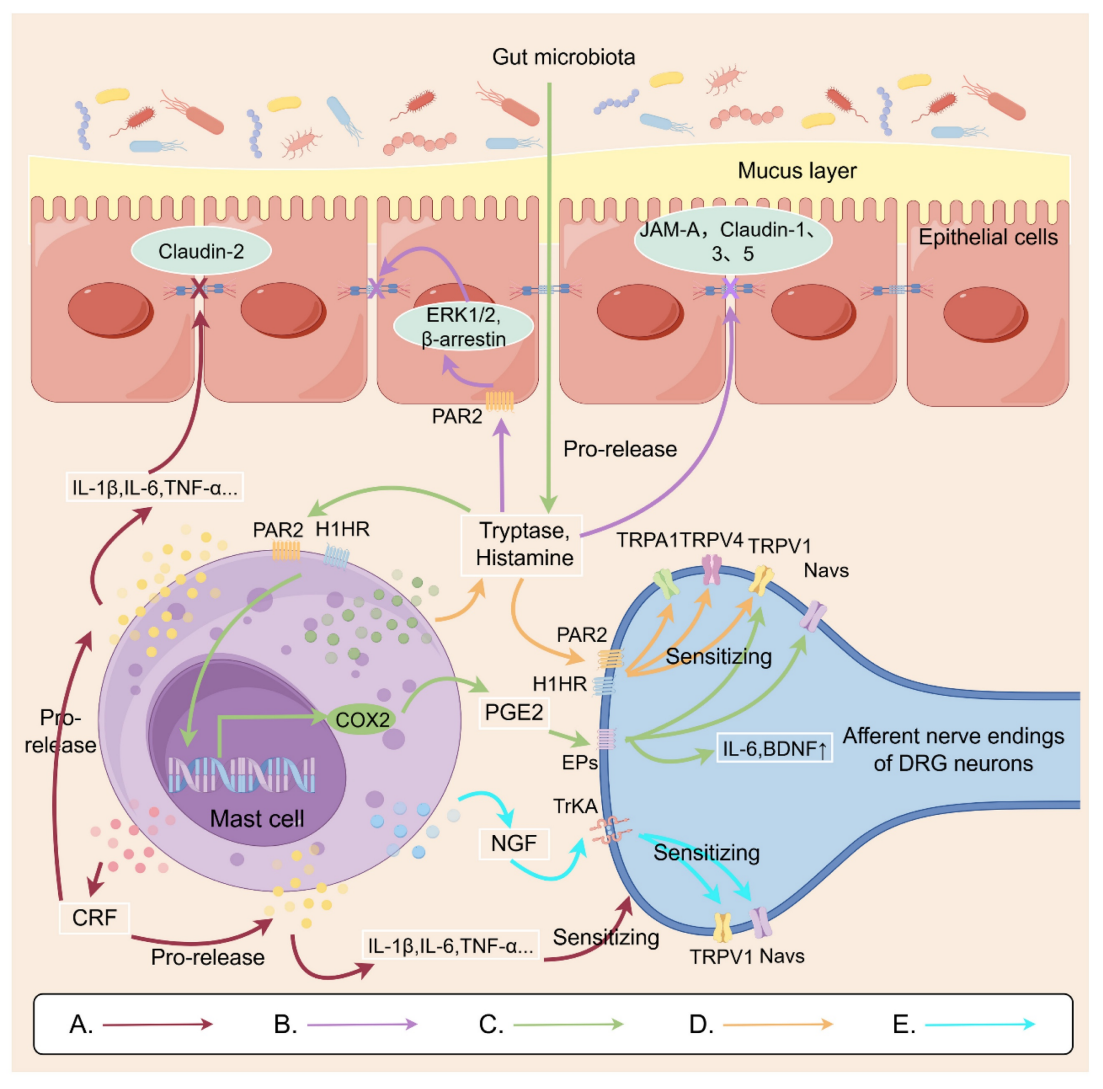
** Possible mechanisms by which mast cells are involved in visceral hypersensitivity.** A: Mast cells secrete the neuropeptide CRF, which modulates macrophage release of inflammatory cytokines. This action can affect the colonic mucosa, reducing expression of the tight junction protein claudin-2. It can also act on DRG nerve endings, increasing sensitivity. B: Mast cells release trypsin, which binds to PAR2 receptors on intestinal epithelial cells. This activates the ERK 1/2 and β-arrestin signaling pathway, affecting the distribution of tight junction proteins on the cell surface. This leads to impaired intestinal barrier function, further contributing to visceral hypersensitivity. C: Histamine and trypsin from the gut microbiota activate mast cells to release PEG2. PEG2 binds to EP receptors on DRG nerve endings, sensitizing TRPV1 and sodium channels. D: Metabolites from gut microbiota induce mast cell degranulation, releasing tryptase and histamine that act on DRG nerve endings to sensitize TRPV1/TRPV4/TRPA1 receptors. E: Mast cell-secreted NGF acts on TrKA in DRG neurons, thereby sensitizing TRPV1 and sodium channels. This Figure was created by Figdraw.

Figure 1 demonstrates the possible mechanisms of MCs involved in VH, which might be a novel promising therapeutic target for IBS or IBD.

#### 3.2.2 Other immunocytes

In IBD patients, there is an increased infiltration of neutrophils and macrophages, which were proved to secret IL-1β, IL-6, TNF-α, PGE2, prokineticin 2 (PROK2) and insulin-like growth factor I (IGF-1). IL-1β, IL-6 and TNF-α lower the thresholds of Nav channels or enhance the expression of TRPV1 channels in the DRG neurons 46, 47, 142-145. The PROK2, an inflammatory cytokine-like molecule, increases intracellular calcium levels in enteric and dorsal root ganglia neurons, which plays a role in IBD caused by trinitrobenzene sulfonic acid (TNBS) 60. IGF-1 enhances TRPV1-mediated membrane currents in DRG neurons and promotes TRPV1 translocation to the cell membrane 146. In addition, patients with IBD and corresponding animal models exhibit elevated expression of transient receptor potential melastatin (TRPM) channels 2, 3, and 8. These channels play a key role in sensitizing DRG neurons and driving VH 147. However, how immune cells influence TRPM expression in IBD remains unclear.

From these contents, VH is greatly influenced by immunocytes, especially in IBD. The effectors secreted from immunocytes mainly increase intestinal permeability, expression of nociceptors, concentration of 5-HT, and so on. For better understand the potential contribution of immunocytes on VH, Table 3 summarizes the main mechanism of inflammatory effectors in the IBS or IBD.

### 3.3 EGCs

In the enteric nervous system, there are plenty of EGCs, which not only support the growth of neurons but also regulate the neural activity. As known, EGCs release NGF to support the growth, survival, and maintenance of neurons 152. However, NGF levels significantly increase during inflammation, and NGF receptor TrkA are extensively coexpressed with TRPV1 in visceral afferents. The abnormal increased NGF not only promotes nerve ending growth, but also potentiates TRPV1 signaling, which might induce visceral hyperalgesia and hypersensitivity 153. Additionally, EGCs activated by inflammatory cytokines in IBD release PGE2, which further sensitizes TRPV1 on afferent nerve endings of DRG neurons via EP4 receptor 56. It is also found that more SP is released in a PLCγ1-dependent manner from EGCs in the IBS model mice. SP, as the first discovered member of the tachykinin family, has long been considered an effector of pain, acting on nociceptive nerve endings and participating in the development of VH 27. Therefore, it indicates that EGCs directly modulate sensibility of sensory neurons via various mechanisms.

Furthermore, Vladimir et al. demonstrated that in dextran sulfate sodium induced colitis, EGCs stimulated by IL-1β release TNF-α to activate macrophages, which could act on the terminals of DRG neurons inducing visceral nociceptor sensitization 62. Additionally, ECGs were found to regulate other types of immune cells, including MCs, antigen-presenting cells, and lymphocytes, which enhanced neuroinflammation, TRPV1-positive neuronal varicosities, and Glia-immunocytes interactions in the IBS to participate in VH 61, 63.

Therefore, the complex functions of EGCs make themselves play an important role in the development of VH.

## 4. Brain nuclei and circuits involved in VH

While the drivers of VH often originate in the periphery, the pain experience is ultimately mediated and amplified by the central nervous system. As known, special brain nuclei are responsible for processing information of various visceral stimulation and producing pain perceptions. There are other different nuclei and neural circuits involved in modulation of visceral pain and generation of negative emotions. Therefore, exploring the central neuromodulation mechanism of VH in patients with intestinal disorders may provide suitable targets for the treatment of the visceral pain of such diseases 154.

The anterior cingulate cortex (ACC) and the paraventricular nucleus of hypothalamus (PVN) of the hypothalamus are extensively involved in the modulation of both ascending pain transmission and descending pain inhibition. Moreover, these regions serve as critical hubs in the neural circuitry responsible for processing emotions and pain signals. This review primarily focuses on potential alterations in these nuclei—particularly the ACC and PVN—in patients with intestinal disorders, as well as their functional roles in such conditions 155.

### 4.1 ACC

The ACC, as a crucial area within the limbic system, is involved in the processing of sensory and emotional components of chronic pain. Functional magnetic resonance imaging studies have shown heightened ACC activation in individuals with IBS 156. Studies showed that chemical and electrical stimulation on ACC enhanced the visceral motor response to colorectal distention (CRD) and nociceptive sensitization in normal or IBS rats, while ACC impairment lessens the response 65. The IBS patients also demonstrated higher activation levels of ACC and greater intensity of pain than healthy control 157. During IBD, ACC exhibits more activation of neuron or microglia in animal model, and shows higher low-frequency fluctuation values and degree centrality values in patients, indicating abnormal brain metabolism within the ACC of IBD patients 64.

These findings collectively demonstrate that the ACC plays an essential role in processing the affective dimension, anticipation, and memory formation of pain. Meanwhile, ACC has a wide range of fiber connections with different brain nuclei (including the substantia nigra, hippocampus, and insular cortex), which are involved in the modulation of emotions and sensations linked to pain 158, 159.

#### 4.1.1 CL-ACC circuit

The central lateral nucleus (CL), positioned between the insular cortex and the striatum with broad links to the cerebral cortex, is essential in the transmission of visceral pain. Xu's experiments utilized neonatal maternal deprivation (NMD)-induced VH mice to evaluate the functional role of nerve fiber projections from CL to ACC in visceral pain processing 66. The results demonstrated that c-Fos expression was significantly increased in the CL of NMD mice, indicating that visceral pain can activate the CL. The activation of glutamatergic neurons in the CL can lead to the activation of those in the ACC, potentially contributing to the development of persistent internal pain. For VH, overexpression of NMDAR and hyperactivation of calcium-calmodulin-dependent protein kinase II α (CaMKIIα) in the postsynaptic density region of the ACC may sensitize the CL-ACC neural circuitry in NMD mice. This was further supported by other experiments showing that an inhibitor targeting NMDAR in glutamatergic neurons of the ACC might be a new point for relieving visceral pain in IBS 67.

#### 4.1.2 MT-ACC circuit

Medial thalamus (MT), the primary relay station for transmitting noxious information to the ACC, has been shown to modulate visceral pain via the MT-ACC pathway 68. The increase in local field potential of rats with VH indicates enhanced synaptic activity at the MT-ACC interface. θ burst stimulation could induce long-term potentiation (LTP) at MT-ACC synapses of normal rats. These findings suggest that electrically induced LTP and VH may share a common mechanism. To test this hypothesis, repeated application of θ-mode stimulation at MT in normal rats enhanced CRD-induced ACC responses and increased visceral pain. The results imply that the MT-ACC pathway might be an important regulating mechanism of visceral pain 69.

### 4.2 PVN and its associated neural circuits

The PVN serves as a critical neuroendocrine hub, releasing several neuropeptides including CRF, arginine vasopressin, oxytocin, and thyrotropin-releasing hormone 72. Its best-characterized role lies in initiating the hypothalamic-pituitary-adrenal (HPA) axis: CRF from the PVN stimulates pituitary adrenocorticotropic hormone secretion, which in turn promotes glucocorticoid release from the adrenal cortex 160. Zhang et al. revealed that administration of CRF-RNAi effectively prevented VH in rats 70. Mechanistic studies indicate that increased CRF elevated glucocorticoid secretion, which might lead to the overexpression of proinflammatory cytokines such as TNF-α, IL-1β, TLR4 et al. 70, 71. Prolonged excessive glucocorticoid exposure was found to cause impairment of hippocampal function, which weakened the inhibitory effect of hippocampus on the HPA axis, leading to sustained release of CRF 161. This vicious CRF- glucocorticoid circle might be an important promoter of VH. These discoveries emphasis the key role of PVN in the regulation of visceral sensory. The PVN also has widely bidirectional fiber connection with brain nuclei, such as lateral septal nucleus (LSV), ventral tegmental area (VTA), BNST, prefrontal cortex (PFC), arcuate nucleus (ARC), which are involved in different functional modulations, including sensory modulation.

#### 4.2.1 PVN-LSV circuit

The LSV is capable of modulating affective behavior and visceral pain. For example, activating the PVN-LSV glutamatergic projections intensifies visceral pain in NMD mice, reversely inhibiting the circuits relieves the visceral pain 73.

#### 4.2.2 PVN-VTA circuit

The VTA is an important center processing reward, addiction, learning, sleep-wakefulness cycles, and so on. The projections of CRF neurons to the VTA are found to be involved in colonic distension-induced pain 74. In IBS model mice, the active CRF neurons significantly increased c-Fos expression and calcium ion activity of VTA glutamatergic neurons, and selective NMDA receptor 2A inhibitor administrated in VTA decreased the number of visceral pain-induced c-Fos positive neurons and attenuated visceral pain 83. Therefore, the paraventricular nucleus of the thalamus (PVT)-VTA circuit might be another molecular target involved in chronic visceral pains.

#### 4.2.3 PVN-PFC circuit

The PFC is the anterior portion of the frontal lobe of the cerebral cortex. The PFC is not only important in executive functions such as planning, problem solving, and social control, but also pain processing, which is dependent on neural circuits with hypothalamus, hippocampus, periaqueductal gray (PAG), amygdala, and other pain-related areas of brain. Recent studies have demonstrated that PVN oxytocin signaling boosts PFC neuronal activity in response to acute pain stimulation. However, in the context of chronic visceral pain, PVN oxytocin signaling to the PFC alleviates both the emotional and sensory dimensions of pain induced by mechanical stimuli and suppresses the development of VH 75.

#### 4.2.4 BNST-PVN circuit

As a part of the extended amygdala, the BNST plays a critical role in stress response, fear memory, and social behavior. It has been reported that the anteroventral BNST (avBNST) directly projects GABAergic and glutamatergic neural fibers to the PVN. The avBNST GABAergic neurons inhibits activity of PVN CRF neuron to alleviate visceral pain. Conversely, glutamatergic inputs from the avBNST to the PVN exert an excitatory effect. In mice prone to VH, an imbalance between two types of inputs was noted, leading to heightened excitability of PVN CRF neurons and increased CRF release, which triggers the HPA axis 71, 76, 77. Therefore, stimulating glutamatergic neurons or suppressing GABAergic neurons within the avBNST-PVN neural pathway promotes VH.

**Figure 2:**
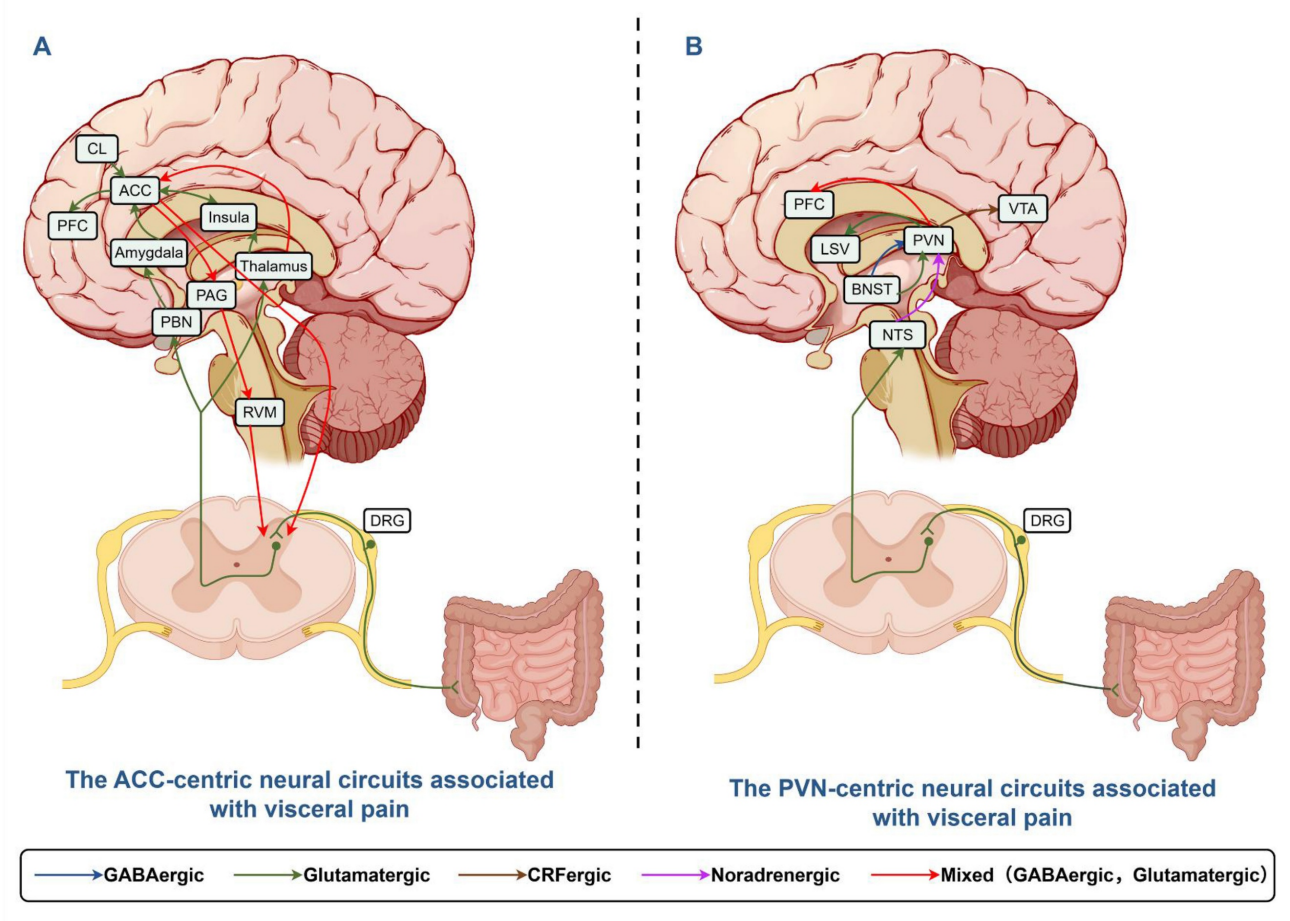
** The ACC-centric and PVN-centric neural circuits associated with visceral pain.** A: Afferent neurons convey signals to DRG and spinal dorsal horn nuclei, projecting directly to thalamus or indirectly to ACC via PBN/Amygdala. ACC modulates spinal neurons through PAG/RVM, integrating BLA/CL/LC/NTS inputs for visceral pain and emotion regulation. B: Afferents transmit signals to DRG, projecting to PVN. BNST sends glutamatergic/GABAergic signals to PVN, which projects to LSV/VTA/PFC for visceral pain modulation. This Figure was created by Figdraw.

The neural circuits of the ACC and PVN involved in the context of visceral pain were summarized and illustrated in Figure 2.

### 4.3 ARC

The ARC is a critical hypothalamus area involved in energy homeostasis and mediating pain sensation. In the ARC of chronic pancreatitis (CP) model rats, expression of cystathionine β-synthetase (CBS) and PKC were significantly upregulated, accompany with higher phosphorylation level of the NMDA receptor GluN2B subunit 78. Microinjection of the CBS inhibitor aminooxyacetic acid into the ARC of CP rats reversed the upregulation of PKC and alleviated VH. The results indicate that CP causes an increase in CBS expression in the ARC, which may play a role in VH by activating NMDA receptor.

NF-κB is another important molecule in the ARC to affect the processing of visceral pain. The selective NF-κB inhibitor pyrrolidine dithiocarbamate alleviated visceral pain in NMD rats 79. G Protein-Coupled Receptor Kinase 6 (GRK6) is a kind of endogenous protein suppressing NF-κB expression, and was found to be low expressed in the ARC of NMD rats, which is believed to be an important modulator of VH.

### 4.4 Rostral ventromedial medulla

The rostral ventromedial medulla (RVM) is a relay station connecting some brain regions like the PAG and amygdala to the dorsal horn of the spinal cord. The RVM dynamically balances pain signals through two types of neurons: ON cells promoting the transmission of pain signals in the spinal cord and OFF cells inhibiting the transmission. ON cells specifically express G protein-coupled estrogen receptor (GPER), which is recognized by estrogen to significantly enhance ON cells activation via the Ca²⁺/PKC pathway. Once activated, these GABAergic ON neurons reduce the inhibitory tone of spinal interneurons, thereby facilitating ascending pain transmission 162. Estrogen also inhibits opioid signaling in ON cells, leading to increased pain and reducing the analgesic effects of morphine 82. In contrast, testosterone exerts sex-dependent effects in the RVM: it downregulates serotonin transporter expression while upregulating 5-HT2A receptor mRNA. These changes are associated with reduced hyperalgesia in female preclinical models, but not in males 163. Collectively, estrogen-mediated facilitation via GPER and opioid interference, together with testosterone-modulated serotonergic signaling, provide a mechanistic basis for the pronounced female predominance observed in functional visceral pain disorders.

### 4.5 Other nuclei

The reuniens (Re) is a component of the ventral midline thalamus, and is associated with diverse cognitive functions, such as working memory and attentional processes. A significant increase of c-Fos-positive neurons, primarily the glutamatergic neurons, was observed following CRD stimulation in the Re of mice with NMD-induced VH, accompanied by an upregulation of 5-HT2B receptor levels in glutamatergic neurons within Re. Then, blocking 5-HT2B receptors in Re led to decreased c-Fos expression and eased visceral pain 80. The findings suggest that enhancing 5-HT2B receptor levels in glutamatergic neurons of Re might be a key factor contributing to VH.

The insula cortex integrates autonomic activity from the viscera and has been referred to as the "visceral brain." In mice with an NMD-induced IBS model or a TNBS-induced IBD model, there was an increase in c-Fos expression in the insula cortex and a boost in excitatory synaptic transmission. Compared with healthy controls, the insula cortex of IBS patients exhibited persistent activation both at rest and during CRD stimulation 155. Further studies show that glutamatergic neurons of the insula cortex are significantly increased by CRD in the neonatal colonic inflammation (NCI)-induced IBS model, and neural signals transmitted from PVT glutamatergic neurons to insula cortex glutamatergic neurons promoted colonic pain in NCI mice 81. These findings suggest that the insula cortex might be involved in the formation of visceral pain.

### 4.6 Developmental windows program stress-responsive brain nuclei and VH

It has been known that the structure and function of mammal brain undergo tremendous changes with development. Based on the brain's plasticity and adaptive capacity, the timing of initial VH is a key determinant of pathogenesis and long-term outcomes. Comparing with the results of stress in adult, NMD primarily simulates severe psychosocial trauma during early life, and particularly activates stress-related regions like PFC, amygdala, hypothalamus, and hippocampus, even the HPA axis. Phenotypic features generated by NMS models include: permanent alterations in central stress circuits (e.g., amygdala, PFC), hyperactivity of the HPA axis with elevated baseline CRF levels, long-term gut dysfunction (permeability, microbiome alterations), more severe and treatment-resistant VH in adulthood. In contrast, adult-onset models typically induce physiological and psychological responses, and produce transient hypersensitivity that resolves more readily, highlighting distinct underlying neuroplasticity mechanisms. These studies suggest that early intervention in children with functional abdominal pain or a history of adversity may prevent the development of chronic, severe clinical symptoms in adulthood 164.

## 5. Gut microbes participate in VH

The human gastrointestinal tract is colonized by a diverse array of microorganisms, encompassing bacteria, viruses, fungi, and protozoa, with bacteria being the most predominant component. Gut microbes are involved in numerous physiological processes, including nutrition, metabolism, defense, and immunity. The microbiota serves not merely as a local factor but as the core biological interface connecting the peripheral gut and the central brain. It drives a complete sensitization loop between the periphery and the central nervous system, thereby forming a “brain-gut” sensitization system. Dysbiosis of the intestinal flora is frequently observed in patients with VH, characterized by altered abundance of specific bacterial populations and an imbalance between beneficial and opportunistic pathogenic bacteria 165.

### 5.1 *Bifidobacterium* and *Lactobacillus* participate in VH by affecting the intestinal barrier

*Bifidobacterium* and *Lactobacillus* are gram-positive bacterial genus, and common probiotics in the human gastrointestinal tract. The strictly anaerobic* Bifidobacterium* belongs to the phylum *Actinobacteria*, and primarily resides in the colon. *Lactobacillus*, a member of the family *Lactobacillaceae*, is either partially anaerobic or microaerophilic and predominantly colonizes the small intestine and stomach. Studies about VH have noted the involvement of the bacteria in both genus, such as *Bifidobacterium longum* (*B. longum*), *Bifidobacterium infantis* (*BCM*), and* Lactobacillus acidophilus* (*LCM*) 88.

**Figure 3:**
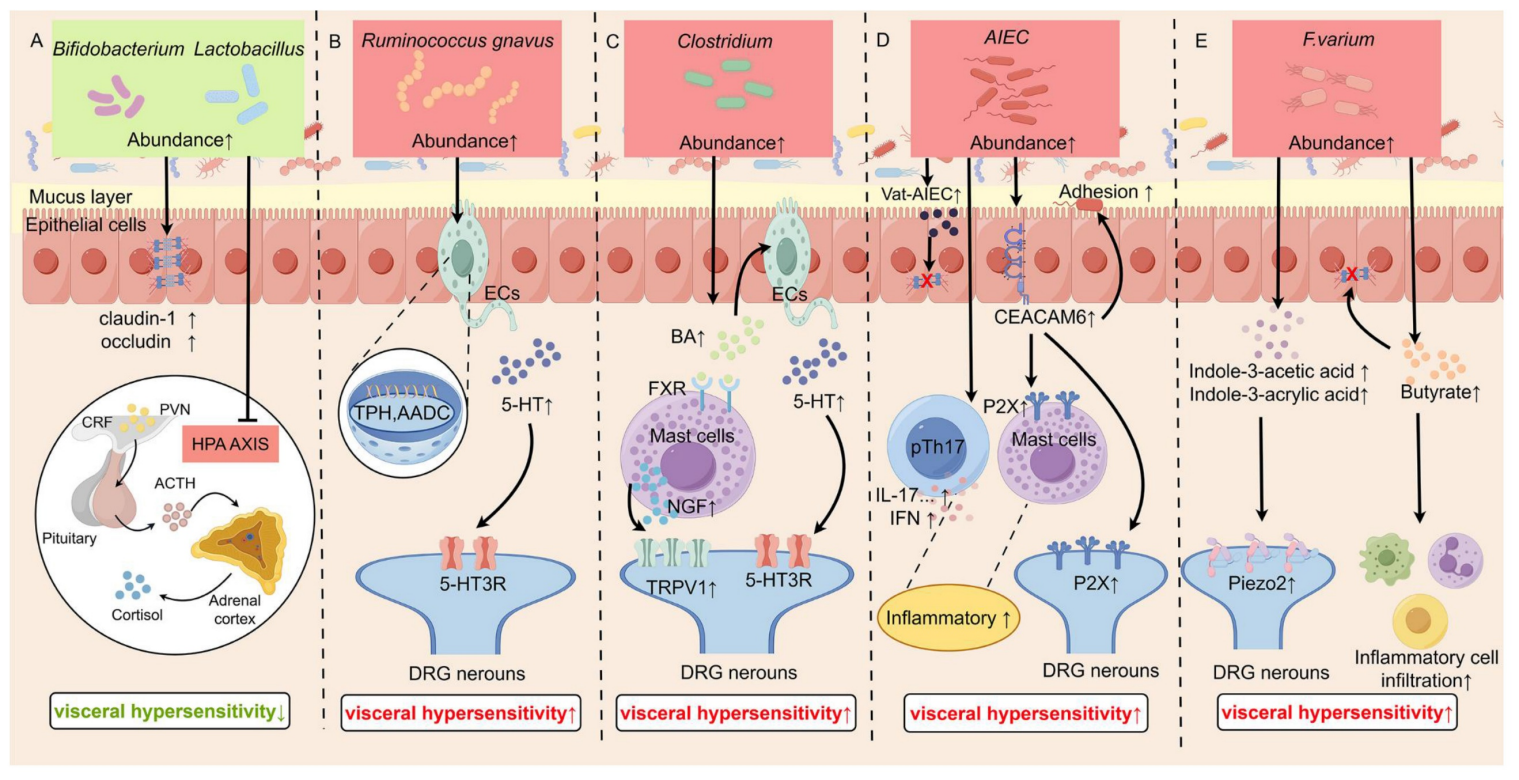
** Mechanisms by which specific gut microbiota modulate visceral hypersensitivity.** A: Probiotics (*Bifidobacterium longum*, *Lactobacillus*) alleviate visceral hypersensitivity by enhancing the intestinal barrier and dampening HPA axis activity. B: *Ruminococcus gnavus* promotes visceral hypersensitivity by increasing intestinal 5-HT synthesis. C: *Clostridium* species exacerbate sensitivity via a bile acid-FXR-NGF axis that upregulates TRPV1 expression on sensory neurons. D: *AIEC* induces hypersensitivity through barrier disruption and activation of mast cells/macrophages via proteases and CEACAM6. E: *F. varium* elevates visceral sensitivity by upregulating Piezo2, impairing the barrier, and promoting inflammatory cell infiltration. This Figure was created by Figdraw.

*B. longum* is one of the major probiotics colonizing the human gastrointestinal tract. In IBS patients, the colonization of *B. longum* in the intestines was significantly reduced compared to that in normal subjects 166. Administration of *B. longum* not only reduced depression scores and alleviated stress in IBS patients, but also reversed the reduction in crypt depth and villus length, decreased intestinal permeability, and restored the proliferation of intestinal mucosal IECs 84, 85. *BCM* and *LCM* were found to normalize the protein expression of claudin-1 and occluding, thereby protecting the intestinal barrier and preventing inflammation 86, 87, 89. Furthermore, it's shown that the combination of *Lactobacillus* and *Bifidobacterium* reduced CRF secretion and HPA axis activity, contributing to recover of VH, anxiety, and depression 90. Therefore, *Bifidobacterium* and *Lactobacillus* exert the function of “probiotics” via various ways, Figure 3A shows their mechanisms for relieving VH.

### 5.2 *Ruminococcus gnavus* participates in VH by affecting peripheral 5-HT levels

*Ruminococcus gnavus* is Gram-positive, anaerobic bacterium that exhibits spherical or spheroidal morphologies, and is involved in digestion and immune regulation 93. It was demonstrated that *Ruminococcus gnavus* was enriched in IBD patients and increased with disease activity 167.

*Ruminococcus gnavus* was found to increased serum 5-HT levels, which was drawn by analyzing the correlation between intestinal flora composition and serum 5-HT levels in IBS-D patients 91. Increased *Ruminococcus gnavus* promotes the conversion of phenylalanine and tryptophan into phenethylamine and tryptamine, which in turn activate protein kinase A (PKA) and CaMKII, leading to an increase in TPH1 and aromatic L-amino acid decarboxylase (AADC), and subsequently causing 5-HT biosynthesis. Then, 5-HT contributes to VH development by acting on the afferent nerve endings of nociceptive DRG neurons (Figure 3B) 92.

### 5.3 *Clostridium* participates in VH by increasing the bile acids excretion

*Clostridium,* an anaerobic Gram-positive bacterium of the *Firmicutes* phylum, participates in various metabolic activities, such as carbohydrate fermentation, protein breakdown, and bile acid (BA) metabolism. About the BA metabolism, experiments in pseudo-germ-free mice colonized with *Clostridium* revealed upregulated expression of Cyp7a1 and Cyp8b1, key enzymes in BA synthesis, thereby enhancing hepatic BA production. Concurrently, reduced ileal Fgf15 expression attenuated farnesoid X receptor (FXR)-mediated BA reabsorption, while increased taurine-conjugated BAs in the intestinal lumen inhibited FXR activation, further diminishing BA reuptake 168.

Researches indicated a positive relationship between elevated levels of fecal BA and the severity of abdominal pain in IBS patients and visceral pain model rats 94, 169, highlighting the critical role of BAs in the pathogenesis of VH. Regarding the mechanisms underlying BA-induced visceral sensitivity, two principal pathways have been proposed. One explanation is that BA activates the FXR on MCs, leading to increased release of NGF. This, in turn, enhances the expression of TRPV1 in DRG neurons, ultimately promoting VH 96. An alternative mechanism suggests that BA can trigger the release of 5-HT from EECs through activation of the Takeda G protein-coupled receptor 5 170.

Therefore, there is a strong association between *Clostridium*-rich microbiota, elevated BA indices, and VH, and Figure 3C shows the mechanisms in a diagram.

### 5.4 Adherent-Invasive *E. coli* participate in VH by increasing intestinal permeability and P2X receptor expression

*Adherent-Invasive Escherichia coli* (*AIEC*) is a subtype of diarrhea-inducing *E. coli* isolated from the enteropathogenic *E. coli* group. This strain exhibits the ability to adhere to IECs and proliferate on the surface of IECs, leading to microvillous lesions. Researches showed that patients with CD have a higher occurrence of *AIEC* in the ileal mucosa, along with unusual expression of carcinoembryonic antigen-related cell adhesion molecules (CEACAM) 5 and CEACAM6 in the ileum 98. Individuals with IBS frequently exhibit increased levels of *AIEC*.

The potential mechanism of VH induced by* AIEC LF82* is complicated (Figure 3D). One reason is that the protein hydrolase Vat-AIEC secreted by AIEC promotes mucin degradation and disrupts the intestinal barrier, thereby increasing intestinal permeability 171. Another reason is the level of CEACAM6 receptor is elevated in patients with IBS and IBD, facilitating the adhesion of *AIEC* to IECs. Additionally, *AIEC* enhances the expression of P2XRs in the colon, which are involved in the formation and transmission of visceral pain 97. Furthermore, *AIEC* was found to activate pTh17 cells *in vivo* or *in vitro*, leading to the production of pro-inflammatory cytokines that promote the development of VH 100.

### 5.5 *F. varium* participates in VH by elevating Piezo2 expression

Chen et al. showed that fecal microbiota transplantation (FMT) from IBS-D patients into germ-free mice induced VH and intestinal motility dysfunction. These mice also exhibited significantly increased Piezo2 expression in the colon and DRG. Through fecal 16S rRNA sequencing, *Fusobacterium varium (F. varium)* was identified as a key bacterial genus. *F. varium* is suggested to elevate Piezo2 levels and increase production of the metabolites indole-3-acetic acid and indole-3-acrylic acid, which can bind to Piezo2. Knocking down Piezo2 alleviated the IBS-D-like symptoms induced by the microbiota transplant 111. Furthermore, *F. varium*, isolated from the colonic mucosa of patients with UC, successfully induced symptom and pathological changes of VH in mice via the metabolic production butyrate, which might disrupt the intestinal barrier and induce release of cytokines 172. Therefore, it indicates that the increased abundance of *F. varium* induces VH by upregulating Piezo2 expression and producing specific ligands (or metabolites) that bind to and activate the target effectors (Figure 3E).

Certainly, there are many other microorganisms involved in the regulation of visceral sensory, but the detailed functioning manners are still unclear.

Figure 3 illustrates the impact of alterations in gut microbiota abundance on visceral sensitivity and their potential underlying mechanisms.

### 5.6 Microbial metabolites modulate VH

Gut microbiota-derived metabolites are key regulators of visceral sensitivity, achieved through their interactions with the host's nervous, immune, and endocrine systems. A balanced metabolic profile supports homeostasis, whereas dysbiosis-induced shifts can promote VH.

### 5.6.1 Short-chain fatty acids (SCFAs) in VH

SCFAs—primarily acetate, propionate, and butyrate—are key metabolites produced from dietary fiber fermentation. Although recognized as contributors to conditions like IBS, their precise roles remain incompletely understood, and clinical associations across studies are inconsistent, presenting an ongoing controversy 108.

Clinical and microbial observations highlight specific patterns. IBS-D patients exhibit elevated fecal propionate and reduced acetate levels, changes positively correlated with an increase in *Prevotella 9* and *Escherichia-Shigella* abundance, suggesting a link to hypersensitivity 101. More broadly, IBS patients often show higher overall SCFA levels and an increased abundance of producer bacteria like *Veillonella* and *Lactobacillus* 102. Notably, the correlation between gut bacterial abundance and SCFA concentration is strongest in IBS-D, with specific taxa such as *Bacteroides plebeius*, *Prevotella sp*, *CAG:1031*, and *Bifidobacterium pseudocatenulatum* being positively associated 103. In summary, these findings solidify the connection between a dysbiotic microbiota, altered SCFA profiles, and the pathophysiology of IBS, particularly the diarrhea-predominant subtype.

Mechanistically, SCFAs influence visceral sensitivity through multiple pathways. Propionate, for instance, can directly activate the vagus nerve via the GPR41 receptor or indirectly stimulate it by promoting 5-HT release from enterochromaffin cells. SCFAs also modulate ion channel function and regulate the release of inflammatory mediators 173. Thus, SCFAs act at the intersection of neural, endocrine, and immune signaling to modulate gut-brain communication.

The role of butyrate is complex and context-dependent. Some studies indicate it promotes hypersensitivity by upregulating neuropeptides (e.g., substance P, CGRP) in dorsal root ganglion neurons 104 or by activating the MAPK-ERK1/2 pathway 105. Conversely, other research demonstrates that butyrate can alleviate hypersensitivity via anti-inflammatory pathways such as AMPK and PPAR-γ 106. This “double-edged sword” effect appears to be dose-dependent: low physiological concentrations support barrier integrity and exert anti-inflammatory effects, while high concentrations overstimulate neuronal receptors, increasing excitability. This concept is supported by animal studies where VH coincided with elevated* Clostridium sensu stricto 1* and fecal butyrate, both of which decreased after probiotic treatment 107. Therefore, the net effect of butyrate is not inherently beneficial or detrimental but is determined by its local concentration and the physiological context, underscoring the nuanced role of microbial metabolites in health and disease.

#### 5.6.2 Dopamine and gut microbiota in VH

Dopamine is a key catecholamine neurotransmitter, with roughly half of its total production occurring in the intestine. Specific gut bacteria, including *Proteus*, *Bacillus subtilis*, and *Corynebacterium*, are known to produce dopamine. Animal studies support this gut-brain link; FMT from specific pathogen-free mice into germ-free mice alleviated anxiety-like behavior and significantly altered dopamine metabolism. However, clinical findings in IBS patients are inconsistent. One study reported elevated dopamine levels in IBS-C patients compared to healthy controls, with no significant change in IBS-D patients 109. Conversely, another study found lower dopamine levels in the serum and urine of IBS patients overall 110. Despite these discrepancies, the collective evidence indicates an association between altered dopamine signaling and IBS, highlighting this pathway as a potential target for therapeutic strategies.

Table 4 summarizes the common gut microbiota alterations in IBS and IBD.

## 6. Comparative pathophysiology: distinct drivers of VH in IBS and IBD

Although visceral pain is a common hallmark in both IBS and IBD, the underlying pathways leading to hypersensitivity differ fundamentally. Clarifying these distinctions is crucial for both mechanistic research and clinical management.

### 6.1 Etiology and inflammatory microenvironment

The primary drivers of VH in IBD and IBS originate from distinct pathological processes. In IBD, hypersensitivity is primarily driven by tissue damage and inflammation. Active intestinal inflammation acts as a prerequisite, wherein pro-inflammatory cytokines (e.g., TNF-α, IL-1β) and infiltrating immune cells directly stimulate and sensitize peripheral nociceptive neurons 178. By contrast, in IBS the process is best characterized as functional and perceptual dysregulation, centered on aberrant visceral perception arising from brain-gut axis dysfunction. This persistent hypersensitive state stems largely from the central nervous system's misinterpretation of normal gut signals. The dysregulation can be initiated by diverse triggers, such as post-infectious immune activation, gut microbiota dysbiosis, or early-life stress. Although overt inflammation is absent, low-grade immune activation and increased intestinal permeability are often observed 179.

### 6.2 Neuro-immune pathways

The mechanisms and primary drivers of neuro-immune communication differ substantially between IBD and IBS. In IBD, the neuro-immune communication is initiated by the inflammatory response. The abnormal activation of innate and adaptive immunity produces a large amount of inflammatory mediators, which act on enteric nerve endings, driving intense peripheral sensitization 180. In contrast, IBS involves a bidirectional interaction between neural system and immune reaction. At the gut level, low-grade immune activation promotes neuronal sensitization via mast cell-neuron axis or mediator such as PAR2, PGE2, histamine and so on 135. Concurrently, central factors including long-term stress, anxiety and depression induce functional and structural plasticity particularly in emotion-sensory integration brain regions such as ACC and PVN. These central changes can, in turn, amplify peripheral immune responses and intestinal permeability. This bidirectional dysregulation creates a persistent vicious cycle that underpins the chronicity of IBS symptoms 181.

### 6.3 Peripheral vs. central contributions

The relative contributions of peripheral and central sensitization to visceral pain differ markedly between IBD and IBS. In IBD, pain during active inflammation is thought to be primarily driven by peripheral sensitization. This process is characterized by a direct lowering of nociceptor thresholds at the site of inflammation, mediated by local inflammatory mediators 182. As the disease progresses to a chronic stage, persistent peripheral nociceptive signaling can induce secondary central sensitization 183. Conversely, in IBS, although peripheral triggers exist, evidence indicates that central sensitization plays a more significant and enduring role 184. This conclusion is supported by functional neuroimaging showing enhanced reactivity in the limbic system and is further corroborated by strong clinical associations with psychiatric comorbidities and stress reactivity, suggesting dysregulation of the HPA axis and descending pain modulatory systems 185.

### 6.4 Clinical and therapeutic implications

The pathophysiological divergence of VH in IBD and IBS carry direct clinical relevance for diagnosis and management. In terms of diagnosis, pain in IBD correlates with the degree of endoscopic inflammation and serum/fecal inflammatory markers (such as C-reactive protein and fecal calprotectin) 186. In contrast, the diagnosis of IBS is based on symptomatic criteria, primarily the Rome criteria, after excluding organic diseases; the biomarkers (e.g., fecal calprotectin) are mainly used to rule out IBD 154. Regarding treatment, the cornerstone of IBD management is the suppression of immune inflammation (e.g., using biologics), and pain often resolves with the control of inflammation. Treatment for IBS, however, focuses on modulating the brain-gut axis, including the use of visceral analgesics (e.g., opioid receptor modulators), a low-FODMAP diet, neuromodulators (e.g., tricyclic antidepressants), and cognitive-behavioral therapy 2, 154, 187.

## 7. Conclusion and Future Perspectives

This article reviews recent studies investigating the pathogenesis of VH, a common symptom in IBS and IBD. It examines the following aspects: 1) the peripheral neural circuits underlying visceral pain, with a focus on the role of intestinal components, including IECs, immune cells, and EGCs in driving peripheral sensitization of nociceptors on DRG neurons; 2) the sensitization of brain nuclei and associated neural circuits; 3) the mechanisms by which microbes contribute to both peripheral and central pathways leading to VH. These three aspects dynamically interact through neural, endocrine, and immune pathways. The gut microbiota affects the functional states of both the intestine and the brain. In turn, the brain modulates gastrointestinal secretion, immunity, and motility, thereby influencing the diversity and composition of the gut microbial community. This complex interaction constitutes the microbiota-gut-brain axis—a highly coordinated and sophisticated system.

There has been a notable shift in therapeutic paradigms from merely alleviating symptoms to implementing targeted interventions that address the underlying pathophysiology of these conditions. This transition is clearly reflected in the growing clinical adoption of various central modulators, such as low-dose amitriptyline and gabapentin, which specifically target central sensitization. Additionally, mast cell stabilizers like sodium cromoglycate are being utilized to tackle immune activation, while 5-HT receptor antagonists, such as alosetron, are employed to modulate enteroendocrine function. Furthermore, microbiota-directed strategies, such as low FODMAP diets and the use of Bifidobacterium longum probiotics, are gaining traction as effective treatment options. The mechanism-targeted therapeutic strategies for VH phenotypes are listed in Table 5, including molecular targets, representative drugs, proposed mechanism, target phenotype, biomarker and clinical evidence status. However, despite these promising developments, significant translational barriers remain. A lack of non-invasive and objective diagnostic tools, together with over-reliance on subjective patient feedback or invasive interventions like colorectal distension, poses challenges to standardization in treatment protocols. Moreover, the inherent heterogeneity of these diseases complicates the ability to target specific mechanisms effectively, as patients may present with varying contributions from intestinal immunity, microbiota, central regulation, or neuroendocrine pathways. Therefore, real-world implementation of these advanced therapeutic strategies necessitates overcoming these obstacles to facilitate the selection of personalized therapies that cater to the unique needs of each patient.

Further research holds substantial promise in these domains: The construction of a cross-species transcriptomic atlas through single-cell analysis of DRG neurons offers key molecular insights. These include nociceptor signatures, species variations, and gene expression correlates of pain and inflammation, which collectively form a consensus framework for exploring the mechanisms of VH. In-depth characterization of neuronal subtypes thereby supports precise etiological identification 200; identifying multi-omics-derived biomarkers to stratify patients by pathophysiological subtype, enabling mechanism-based targeting; developing novel therapeutics against validated molecular targets; and optimizing combination strategies (pharmacological, dietary, psychosocial) to leverage synergistic effects. VH management will likely remain chronic, but the definitive endpoint—accurate diagnosis, prevention, and personalized care—remains achievable with continued innovation. Collectively, these advancements will shift the paradigm of VH from a poorly defined symptom cluster to a precisely characterized, mechanism-driven clinical entity.

## Figures and Tables

**Table 1:** Confidence and consistency of evidence for key pathways

Pathway/ Mechanism	Representative studies and dominant Evidence Source	Confidence in Human Pathophysiology	Consistency Across Studies
Enterocytes and L cells release ATP/UTP/ADP upon activation, which act on P2X3/7 and P2Y receptors to promote VH	James R F Hockley et al. (animal models) 20 Annemie Deiteren et al. (animal models) 21 Olena A Petrushenko et al. (*ex vivo* studies) 22 Alexander Eser et al. (human *ex vivo* studies) 23 Van B Lu et al. (animal models) 24	Low-Medium	High
Enterocyt and L cells, upon activation, release Glu to promote VH	Michelle Y Meng et al. (animal models) 25 Qingqing Qi et al. (animal models and human studies) 26	Low-Medium	High
Activated enterocytes release BDNF to promote VH	Peng Wang et al. (animal models and human *ex vivo* studies) 27 Yu Zhang et al. (human *ex vivo* studies) 28	Low-Medium	High
CRF in the intestine can promote VH by acting on CRFR1, while acting on CRFR2 inhibits VH.	K Saito Nakaya et al. (animal models) 29 Seth Sweetser et al. (Human RCT) 30 Eloísa Salvo-Romero et al. (human *ex vivo* studies) 31	High	Medium
5-HT (primarily derived from ECs) participates in VH by acting on 5-HT3R	Cesare Cremon et al. (human e*x vivo* studies) 32 James R Bayrer et al. (animal models) 33 Rui Fu et al. (human *ex vivo* studies) 34 Satish S C Rao et al. (human RCT) 35 Ghazaleh Mohammadian et al. (human *ex vivo* studies) 36	High	Medium
Piezo1 and Piezo2 promote VH by acting on EC and DRG	Constanza Alcaino et al. (animal models) 37 Erika Sugisawa et al. (animal models) 38 Zili Xie et al. (animal models) 8	Low	High
The GUY2C pathway regulates VH	Joshua R Barton et al. (*Ex vivo* and animal models) 39 Kunwar Shailubhai et al. (animal models) 40	Low	High
IL-33 acts on MCs to promote VH	Zhiping Wei et al. (animal models) 41	Low	Low
IL-1β participates in VH by promoting intestinal inflammation and disrupting the intestinal barrier.	Tsukasa Nozu et al. (animal models) 42	Low	High
IL-6 promotes VH	Sven Benson et al. (human RCT) 43 Maria M Buckley et al. (animal models) 44 Emmeline Salameh et al. (animal models) 45	Medium-High	High
TNF-α promotes VH	Katie H Barker et al. (animal models) 46 Sagi Gudes et al. (animal models) 47 Sven Benson et al. (human RCT) 43	Medium-High	High
NGF from sources such as MC and EGC promotes VH by facilitating the growth of nerve endings or sensitizing neurons	Giovanni Dothel et al. (human *ex vivo* studies) 48 Yan Chen et al. (animal models) 49	Medium	High
Tryptases promote VH by acting on PAR2 through mechanisms such as disrupting the intestinal barrier.	Silvia Amadesi et al. (animal models) 50 Claire Jacob et al. (human *ex vivo* studies) 51 Tamira K Klooker et al. (human RCT) 52	High	Medium
PGE2 derived from cells such as MC and EGC promotes VH	Gintautas Grabauskas et al. (animal models) 53 Jun Gao et al. (animal models) 54 Karem Awad et al. (human *ex vivo* studies) 55 Wilmarie Morales-Soto et al. (animal models) 56	Low-Medium	High
Histamine promotes VH by acting on H1HR and H4HR.	Annemie Deiteren et al. (animal models) 57 D Balemans et al. (human *ex vivo* studies) 58 Lisse Decraecker et al. (human RCT) 59	High	High
Proke2 promotes VH	Robert P Watson et al. (animal models) 60	Low	Low
EGC interacts with other immune cells to promote VH	Felipe Meira de-Faria et al. (human *ex vivo* studies) 61 Vladimir Grubišić et al. (animal models) 62 Ninotchska M Delvalle et al. (animal models) 63	Low-Medium	High
The activation of ACC (glutamatergic and microglia, etc.) promotes VH	Ling Yang et al. (human cross-sectional studies) 64 Zhijun Cao et al. (animal models) 65	Medium	High
The activation of the CL-ACC circuit promotes VH	Qiya Xu et al. (animal models) 66 Ying Xiao et al. (animal models) 67	Low	High
The activation of MT-ACC promotes VH	Jun Wang et al. (animal models) 68 Ying Li et al. (animal models) 69	Low	High
Activation of CRF neurons in PVN promotes VH	Gongliang Zhang et al. (animal models) 70 Si Ting Huang et al. (animal models) 71 Catherine S Hubbard et al. (human RCT) 72	Medium-High	High
The activation of PVN-LSV promotes VH	Yong Chang Li et al. (animal models) 73	Low	Low
The activation of PVN-VTA (CRF) promotes VH	Ning Ning Ji et al. (animal models) 74	Low	Low
The activation of PVN-PFC (oxytocin) inhibits VH	Yaling Liu et al. (animal models) 75	Low	Low
The inhibition of GABAergic projections and activation of glutamatergic projections from BNST-PVN promote VH	Si Ting Huang et al. (animal models) 76 Yu Song et al. (animal models) 77	Low	Low
Activation of NMDA receptors in ARC promotes VH	Hang Zheng et al. (animal models) 78	Low	Low
Downregulation of GRK6 in ARC leads to upregulation of NF-κB, which promotes VH	Xin Li et al. (animal models) 79	Low	Low
Upregulation of glutamate neurons 5-HT2B receptors in Re promotes VH	Di Li et al. (animal models) 80	Low	Low
Activation of the insular cortex promotes VH	Fu-Chao Zhang et al. (animal models) 81	Low	Low
Estrogen in RVM promotes VH through GPER	Yingfu Jiao et al. (animal models) 82	Low	Low
The activation of VTA promotes VH	Meng-Ge Li et al. (animal models) 83	Low	Low
*B.longum* inhibits VH	A.P.Allen et al. (human clinical controlled trail) 84 Chunhua Zhou et al. (animal models) 85	Medium	High
*BCM* and* LCM* inhibit VH	Shuangshuang Guo et al. (*ex vivo* studies) 86 Erin D Lewis et al. (human RCT) 87 Christopher J Martoni et al. (human RCT) 88 Huan Wang et al. (animal models) 89 Afifa Ait-Belgnaoui et al. (animal models) 90	High	High
*Ruminococcus gnavus* promotes VH	Lixiang Zhai et al. (animal models) 91 Lijuan Han et al. (human cohort studies) 92 Fernanda Cristofori et al. (human RCT) 93	High	High
*Clostridium* promotes VH by increasing BA metabolism	Antal Bajor et al. (human cross-sectional studies) 94 Ling Zhao et al. (human case-control studies) 95 Wen Ting Li et al. (animal models) 96	Medium	High
*Adherent-Invasive E. coli* promotes VH	Amandine Lashermes et al. (animal models) 97 Arlette Darfeuille-Michaud et al. (human ex vivo studies) 98 Franck Carbonnel et al. (human RCT) 99 Moira Paroni et al. (human *ex vivo* studies) 100	High	High
SCFAs regulate VH (conflicting results)	Qing Hua Sun et al. (human case-control studies) 101 C Tana et al. (human case-control studies) 102 Andrea S Shin et al. (human case-control studies) 103 Yi Xing et al. (*Ex vivo* studies) 104 Dabo Xu et al. (animal models) 105 Tsukasa Nozu et al. (animal models) 106 Yingjie Li et al. (animal models) 107 Erfeng Li et al. (human RCT) 108	High	Low
Microbial metabolite dopamine regulates VH (conflicting results)	Cezary Chojnacki et al. (human *ex vivo* studies) 109 Ammar Hassanzadeh Keshteli et al. (human case-control studies) 110	Medium	Low
*F.varium* promotes VH by increasing Piezo2	Haonan Zheng et al. (animal models) 111	Low	Low

Grading Criteria: Confidence: High = Supported by multiple, robust human clinical studies; Medium = Supported by strong animal data and preliminary/mechanistic human evidence; Low = Primarily based on preclinical data with minimal direct human evidence. Consistency: High = Findings are replicated across independent studies; Medium = General agreement exists with some conflicting reports; Low = Evidence is limited or findings are contradictory. Detailed mechanistic descriptions and key references are provided in the main text.

**Table 2:** Intestinal epithelial cell-derived substances participate in visceral hypersensitivity by sensitizing nociceptors

Mediator	Receptors	Source/ Location	Specific Mechanism	Primary Associated Phenotypes	Citation
ATP	P2X3 P2X7	Enterocyte L cell	Enhancing calcium responses sensitizes TRPV1 on DRG neurons Regulating the release of IL-1β	IBS IBD(UC) PI-IBS	21, 22, 24, 113, 126
UTP	P2Y2, P2Y4	Enterocyte	Enhance action potentials induced by electrical stimulation, promoting cell membrane depolarization	-	20
ADP	P2Y1, P2Y12, P2Y13	Enterocyte	Increase action potential discharge	-	20
Glu	NMDAR AMPAR	Enterocyte L cells	Stimulate DRG neurons; Promotes the release of SP and CGRP from DRG neurons; Promotes the release of BDNF from enterocytes.	IBS	12, 28, 114, 125
trypsin-3	PAR2	Enterocyte	Signal to human submucosal enteric neurons	IBS, IBD	115
CRF	CRFR1 CRFR2	Enterocyte	Activation of CRFR1 leads to increased intestinal permeability, while activation of CRFR2 exerts an antagonistic effect. Indirectly promotes the inflammatory response	IBS	116, 117
IL-33	ST2	Enterocyte	Activating mast cells and disrupting the intestinal barrier.	IBD(UC)	41
5-HT	5-HT3R	ECs	Activating submucosal neurons Sensitizing TRPV1 and TRPV4 on DRG neurons.	IBS	33, 121, 122
Piezo2	-	Enterocyte ECs DRG	Promoting the release of 5-HT from ECs; Mediating VH through TRPV1 on DRG neurons.	IBS, IBD IBS, IBD	37, 38, 120 8

**Table 3:** Various immune effectors participate in visceral hypersensitivity

Mediator	Source	Receptors on DRG sensory neurons	Possible mechanisms	Primary Associated Phenotypes	Citation
IL-1β	MC neutrophil macrophage	IL-1R	Promoting intestinal inflammation and disrupting intestinal homeostasis	IBD, IBS	42, 148, 149
			Sensitizing TRPV1	IBD	145
IL-6	MC neutrophils macrophage	IL-6R	Participates in VH by interacting with CRF, increasing intestinal permeability	IBS	43, 44, 142, 149
			Lower the thresholds of Nav channels	IBD	
TNF-α	MC neutrophil macrophage	TNFR	p38MAPK-dependent manner enhances Nav 1.8, Nav 1.9	IBD	47, 149
			p38 MAPK-dependent way to sensitize TRPV1	IBS, IBD	46
NGF	MC	TrkA	Enhancement of Nav 1.7 Sensitization of TRPV1	IBS-D IBD	140 49
		NTRK1	Enhancement of GAP43 transcription to promote neuronal growth	IBD	48
Tryptases	MC	PAR2	Sensitization of TRPV1 in a PKC-dependent manner	IBD	50
			Disruption of the intestinal barrier	IBS, IBD	51, 138, 139
			Promotes the release of PGE2	IBS	53
PGE2	MC Neutrophil Macrophage	EPs	Enhancement of TRPV1 and sodium channels	IBS-D	53, 135
			Promoting VH by elevating mucosal 5-HT levels	IBS-D	54
			Increasing intestinal permeability	IBS-M	55
CRF	MC Eosinophil Enterocyte	CRFR1	Autocrine-promoted MC activates degranulation to release various mediators; Increasing intestinal permeability.	IBS	31, 116, 141
Histamine	MC	H1HR H4HR	Sensitizes TRPV1, TRPA1, TRPV4	IBS IBD (UC)	57, 58
IGF-1	Macrophage	IGF-1R	Sensitization of TRPV1 by SNARE-dependent cytotoxicity	IBD	150
PROK2	Neutrophil Macrophage	PKR1 PKR2	Increases intracellular calcium levels in cultured enteric and DRG neurons Stimulates intrinsic neuronally mediated ion transport in ileal mucosa	IBD (UC)	60
Unclear	Unclear	TRPM2 TRPM3 TRPM8	Increase expression of TRPM2, TRPM3, TRPM8 in the DRG neurons	IBD	147, 151

**Table 4:** Common alterations of gut microbes in IBS and IBD

Type of disease	Gut microbes	Change in abundance	Mechanisms leading to visceral hypersensitivity	Citation
IBS	*Lactobacillus*	lower	Affecting the intestinal barrier	174
IBS	*Lactobacillus*	Higher	Increases lactic acid levels, leading to increased synthesis of SCFAs	102
IBS	*Veillonella*	Higher	Increase SCFA levels	102
IBS	*Hungatella*	Higher	unknown	174
IBS	*Ruminococcus gnavus*	Higher	Promotes peripheral 5-HT synthesis	174
IBS	*Clostridium leptum*	Higher	Promotes BA synthesis	95
IBS	*Clostridium sensu stricto 1*	Higher	Increase butyrate levels	107
IBS	F.varium	Higher	Increase the Piezo2 level	111
IBS	Prevotella 9	Higher	Increase levels of propionic acid	101
IBD (UC)	*Bifidobacterium*	lower	Affecting the intestinal barrier	175
IBD (UC&CD)	*Ruminococcus*	Higher	intestinal inflammation	176
IBD (UC&CD)	*Escherichia coli*	Higher	Increased intestinal permeability and P2X receptor expression	177

**Table 5:** Mechanism-targeted therapeutic strategies for visceral hypersensitivity phenotypes

Molecular Targets	Representative drugs	Proposed Mechanism in VH	Target Phenotype / Biomarker	Clinical Evidence Status
Histamine H1 Receptor	Ebastine	Blocks histamine-mediated activation and sensitization of visceral afferent nerves	IBS patients with evidence of mast cell infiltration and mediator release (e.g., elevated mucosal tryptase).	Phase II Trials 59
	Ketotifen	Stabilizes mast cells, reduced number and decreased activity of mast cells in the intestinal mucosa, especially in the terminal ileum.		Phase II Trials 188
Guanylate Cyclase-C (GC-C)	Linaclotide, Plecanatide	Increases extracellular cGMP, stimulating fluid secretion and reducing nociceptor activity	IBS-C; Patients with constipation-predominant symptoms.	Phase III Trials 189, 190
5-HT3 Receptor	Alosetron, Ondansetron	Antagonizes serotonin-induced activation of visceral sensory pathways, slows colonic transit, allows increased water reabsorption and stool formation.	IBS-D patients; potentially those with elevated mucosal 5-HT availability.	Approved 191
Tryptase (Mast Cell)	Future target	Inhibits tryptase, a key mast cell mediator that activates PAR2 on sensory neurons, disrupting a primary pathway of neuro-immune activation.	A mast-cell-dominant subtype of IBS or IBD-with-IBS symptoms.	Preclinical evidence 192
Bile Acid Modulators	Colesevelam	Colesevelam binds excess BA in the colon, reducing their secretagogue and pro-motility effects, thereby improving stool consistency and avoiding steatorrhoea in patients with IBS-D. It increases the delivery of total and secondary bile acids to stool, enhances hepatic bile acid synthesis, and upregulates colonic mucosal expression of genes involved in regulating BA, farnesoid X (FXR), and GPBAR1 receptors.	IBS-D patients with bile acid malabsorption (BAM).	Phase II Trials 193
P2X3 Antagonist	Gefapixant, Eliapixant	Antagonizing the binding of ATP to P2X3 receptors in the intestine reduces the transmission of visceral discomfort signals to the central nervous system.	-	Due to taste-related side effects of the drug, its clinical development has been discontinued, necessitating the development of a new generation of medications. 194
Microbial Modulation Therapy	Low FODMAP diet	Reduce short-chain carbohydrates that are difficult for the small intestine to absorb and prone to fermentation, thereby lowering intestinal osmotic pressure and gas production.	For all subtypes of IBS patients	Phase II Trials 195
Microbial Modulation Therapy	Supplement specific probiotics	Competing with and excluding harmful bacteria to reduce their survival space, strengthening the intestinal barrier and regulating immunity.	IBS: For patients seeking complementary therapies to alleviate symptoms IBD (UC): Maintenance during the remission period	196
	Probiotic *Lactobacillus BD7807*	Enhanced intestinal barrier function by increasing MUC2 protein, antimicrobial peptide and tight junction gene expression. Increased abundance of beneficial gut bacteria and elevated levels of SCFAs in the gut.		Phase II clinical trial 197
	*Lactobacillus plantarum 299V*	Inhibits the adhesion of pathogenic bacteria, reduces colonic gas production, and modulates immune responses.		Phase II clinical trial 198
	*Bifidobacterium bifidum MIMBb75*	Enhance intestinal barrier function, downregulate intestinal TRPV1 expression and activation and modulate immune responses.		Phase III clinical trial 199

## Abbreviations

5-HT: 5-hydroxytryptamine
AADC: aromatic L-amino acid decarboxylase
ACC: anterior cingulate cortex
AIEC: Adherent-Invasive Escherichia coli
AMPAR: α-amino-5-hydroxy-3-methyl-4-isoxazolepropionic acid receptor
avBNST: anteroventral BNST
B. longum: Bifidobacterium longum
BA: bile acids
BCM: Bifidobacterium infantis
BDNF: brain-derived neurotrophic factor
BNST: bed nucleus of the stria terminalis
CaMKIIα: calcium-calmodulin-dependent protein kinase II α
CBS: cystathionine β-synthetase
CD: Crohn's disease
CEACAM: carcinoembryonic antigen-related cell adhesion molecules
CGRP: calcitonin gene-related peptide
cGMP: cyclic guanosine monophosphate
CL: central lateral nucleus
CP: chronic pancreatitis
CRD: colorectal distension
CRF: corticotropin-releasing factor
CRFR1: corticotropin-releasing factor receptor 1
DRG: dorsal root ganglion
ECs: enterochromaffin cells
EECs: enteroendocrine cells
EGCs: enteric glial cells
FMT: fecal microbiota transplantation
FXR: farnesoid X receptor
Glu: glutamate
GPER: G protein-coupled estrogen receptor
GUCY2C: Guanylyl Cyclase C
H1HR: histamine receptor
HPA: hypothalamic-pituitary-adrenal
IBD: inflammatory bowel disease
IBS: irritable bowel syndrome
IEC: intestinal epithelial cell
IGF-1: insulin-like growth factor-1
IL: intralaminar nucleus
JAM-A: junctional adhesion molecule-A
*LCM*: *Lactobacillus acidophilus*
LSV: lateral septal nucleus
LTP: long-term potentiation
MCs: mast cells
MT: medial thalamus
NA: norepinephrine
NCI: neonatal colonic inflammation
NGF: nerve growth factor
NMD: neonatal maternal deprivation
NMDAR: N-methyl-D-aspartate receptors
NTRK1: neurotrophic tyrosine kinase receptor type 1
P2RX3, P2RX4, P2RX6 and P2RX7: Purinergic receptors P2X 3, 4, 6 and 7
PAG: periaqueductal gray
PAR2: protease-activated receptor 2
PBN: parabrachial nucleus
PFC: prefrontal cortex
PGE2: prostaglandin E2
PKA: protein kinase A
PKC: protein kinase C
PROK2: prokineticin 2
PVN: paraventricular nucleus
PVT: paraventricular nucleus of the thalamus
Re: reuniens
RVM: rostral ventromedial medulla
SANRA: scale for the assessment of narrative review articles
SNARE: soluble N-ethylmaleimide-sensitive factor attachment protein receptor
SP: substance P
TLR4: toll-like receptor 4
TNBS: trinitrobenzene sulfonic acid
TRP: transient receptor potential
TRPM: transient receptor potential melastatin
UC: ulcerative colitis
VTA: ventral tegmental area
VH: visceral hypersensitivity
